# Supplementary material for: Transmission Dynamics of Torque Teno Sus Virus 1 (TTSuV1) Between Wild and Farmed Pigs: A Molecular Tool for Monitoring Cross-Population Spillover in Swine
Source: Microorganisms. 2025 Dec 3;13(12):2751. doi: 10.3390/microorganisms13122751 (PMC12735655; doi:10.3390/microorganisms13122751)
Supplement: Supplementary file 1 [file microorganisms-13-02751-s001.zip › microorganisms-3952846-supplementary.pdf]

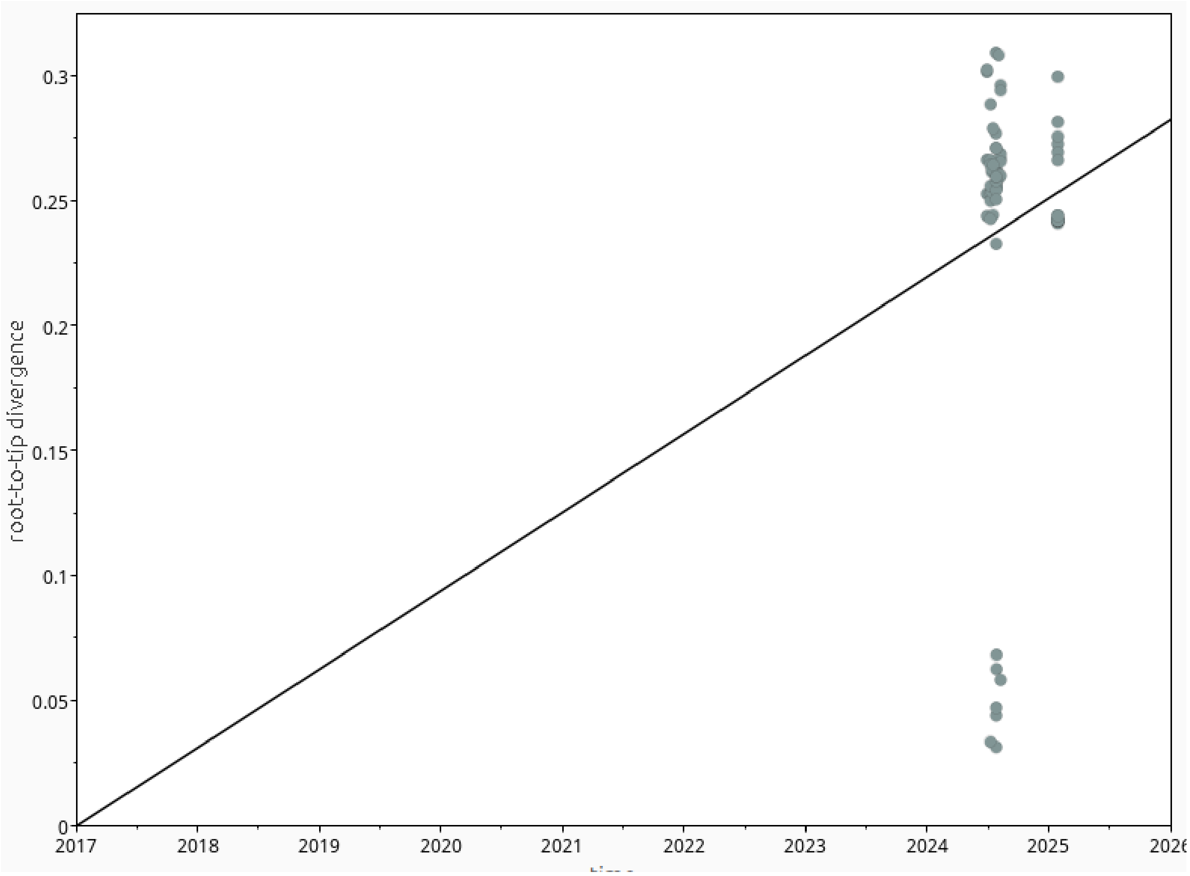

Supplementary Figure S1. Plot of Root-to-tip divergence against sampling dates by TempEst.

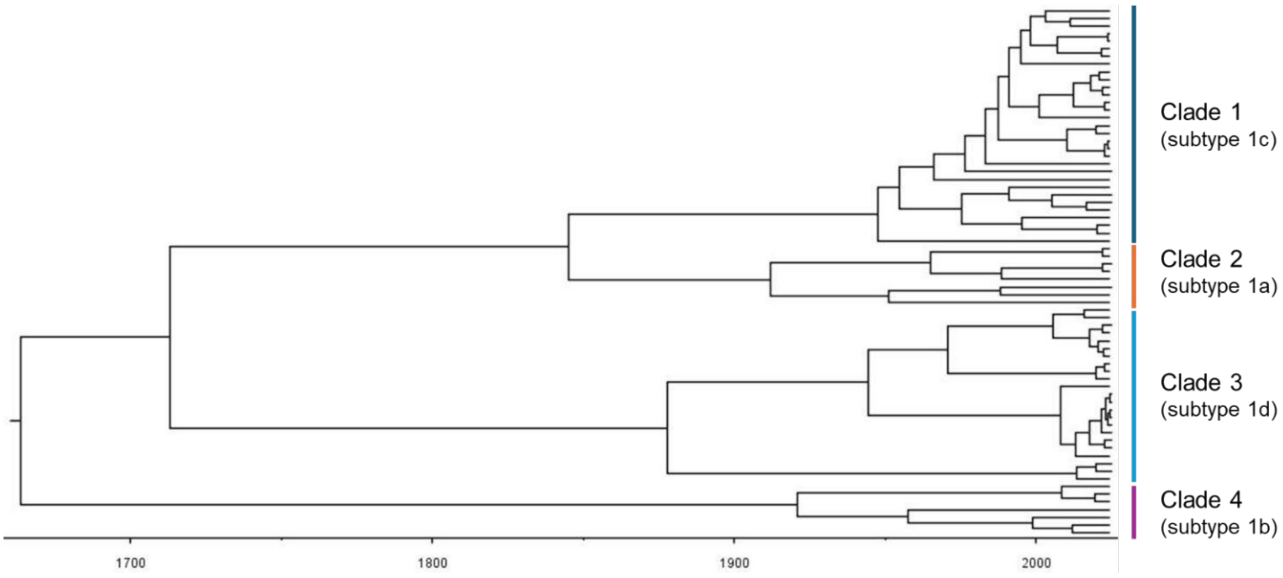

Supplementary Figure S2. Maximum clade credibility (MCC) tree of 69 TTSuV1 sequences generated using BEAST 2 under a relaxed lognormal clock and constant population size model. The tree is time-scaled, with branch lengths representing estimated dates of divergence. The four major clades, corresponding to TTSuV1 subtypes a-d, are color-coded and labeled.

Supplementary Table S1. Prevalence of Torque teno sus virus 1 (TTSuV1) among pig farms in Arkansas, USA.

Farm	# of samples	# of positives (prevalence)
Source farm	49	49 (100%)
Farm A	10	10 (100%)
Farm B	10	10 (100%)
Farm C	9	9 (100%)
Farm D	10	8 (80%)

Supplementary Table S2. Torque teno sus virus 1 (TTSuV1) sequences used in this study.

Isolate name	Accession No.	Host	Country	Source
SUSC-1	PX389932	Domestic	USA	This study
SUSC-2	PX389933	Domestic	USA	This study
SUSC-3	PX389934	Domestic	USA	This study
SUSC-4	PX389935	Domestic	USA	This study
SUSC-5	PX389936	Domestic	USA	This study
SUSC-11	PX389937	Domestic	USA	This study
SUSC-12	PX389938	Domestic	USA	This study
SUSC-14	PX389939	Domestic	USA	This study
SUSC-15	PX389940	Domestic	USA	This study
SUSC-16	PX389941	Domestic	USA	This study
SUSC-17	PX389942	Domestic	USA	This study
SUSC-18	PX389943	Domestic	USA	This study
SUSC-19	PX389944	Domestic	USA	This study
SUSC-20	PX389945	Domestic	USA	This study
SUSC-22	PX389946	Domestic	USA	This study
SUSC-26	PX389947	Domestic	USA	This study
SUSC-28	PX389948	Domestic	USA	This study
SUSC-31	PX389949	Domestic	USA	This study
SUSC-32	PX389950	Domestic	USA	This study
SUSC-33	PX389951	Domestic	USA	This study
SUSC-34	PX389952	Domestic	USA	This study
SUSC-45	PX389953	Domestic	USA	This study
SUSC-50	PX389954	Wild	USA	This study
SUSC-51	PX389955	Wild	USA	This study
SUSC-52	PX389956	Wild	USA	This study
SUSC-53	PX389957	Wild	USA	This study
SUSC-55	PX389958	Wild	USA	This study
SUSC-59	PX389959	Wild	USA	This study
SUSC-61	PX389960	Wild	USA	This study
SUSC-64	PX389961	Wild	USA	This study
SUSC-68	PX389962	Wild	USA	This study
SUSC-70	PX389963	Wild	USA	This study
SUSC-76	PX389964	Wild	USA	This study
SUSC-78	PX389965	Wild	USA	This study
SUSC-80	PX389966	Wild	USA	This study
SUSC-82	PX389967	Wild	USA	This study
SUSC-83	PX389968	Wild	USA	This study
SUSC-84	PX389969	Wild	USA	This study
SUSC-85	PX389970	Wild	USA	This study
SUSC-86	PX389971	Wild	USA	This study
SUSC-87	PX389972	Wild	USA	This study

SUSC-88	PX389973	Wild	USA	This study
SUSC-89	PX389974	Wild	USA	This study
SUSC-90	PX389975	Wild	USA	This study
SUSC-92	PX389976	Wild	USA	This study
SUSC-94	PX389977	Wild	USA	This study
SUSC-96	PX389978	Wild	USA	This study
SUSC-99	PX389979	Wild	USA	This study
SUSC-105	PX389980	Wild	USA	This study
SUSC-107	PX389981	Wild	USA	This study
SUSC-108	PX389982	Wild	USA	This study
SUSC-111	PX389983	Wild	USA	This study
SUSC-113	PX389984	Wild	USA	This study
SUSC-114	PX389985	Wild	USA	This study
SUSC-116	PX389986	Wild	USA	This study
SUSC-117	PX389987	Domestic	USA	This study
SUSC-119	PX389988	Domestic	USA	This study
SUSC-124	PX389989	Domestic	USA	This study
SUSC-125	PX389990	Domestic	USA	This study
SUSC-126	PX389991	Domestic	USA	This study
SUSC-128	PX389992	Domestic	USA	This study
SUSC-129	PX389993	Domestic	USA	This study
SUSC-130	PX389994	Domestic	USA	This study
SUSC-131	PX389995	Domestic	USA	This study
SUSC-132	PX389996	Domestic	USA	This study
SUSC-135	PX389997	Domestic	USA	This study
SUSC-137	PX389998	Domestic	USA	This study
SUSC-138	PX389999	Domestic	USA	This study
SUSC-139	PX390000	Domestic	USA	This study
SUSC-140	PX390001	Domestic	USA	This study
SUSC-141	PX390002	Domestic	USA	This study
SUSC-143	PX390003	Domestic	USA	This study
SUSC-146	PX390004	Domestic	USA	This study
SUSC-147	PX390005	Domestic	USA	This study
SUSC-148	PX390006	Domestic	USA	This study
SUSC-149	PX390007	Domestic	USA	This study
SUSC-150	PX390008	Domestic	USA	This study
SUSC-153	PX390009	Domestic	USA	This study
SUSC-155	PX390010	Domestic	USA	This study
PTTV1a-VA	GU456383	Domestic	USA	NCBI
PTTV1b-VA	GU456384	Domestic	USA	NCBI
Sd-TTV31	AB076001	Domestic	Japan	NCBI
swSTHY-TT27	GQ120664	Domestic	Canada	NCBI
TTV1_1914	GU570200	Domestic	Spain	NCBI
TTV1_AUS05	JF451530	Domestic	Australia	NCBI

TTV1_ESP93	JF451553	Domestic	Spain	NCBI
TTV1_G21	GU570201	Domestic	Spain	NCBI
TTV1_USA07	JF451557	Domestic	USA	NCBI
TTV1_USA11	JF451447	Domestic	USA	NCBI
TTV2_USA12	JF451824	Domestic	USA	NCBI

Supplementary Table S3. Model comparison using path sampling for combinations of clock and population models in BEAST2. Marginal likelihoods and Bayes factors (BF) are shown relative to the best-fitting model.

**Clock Model**	**Population Model**	**Marginal Likelihood (log)**	**Log Bayes Factor (BF)**
Relaxed Clock LogNormal	Constant	−4354.97	0.00 (Best)
Relaxed Clock LogNormal	Exponential Growth	−4356.25	−1.28
Relaxed Clock Exponential	Constant	−4381.69	−26.72
Relaxed Clock Exponential	Exponential Growth	−4381.61	−26.64

Supplementary Table S4. Negative binomial regression models for assessing the association between transmission steps and geographic distance.

Model	Variable	IRR$	95% CI*	*P*-value
Wild-wild pairs	Intercept	43.53	[37.06, 51.48]	0.000
	Geographic distance	1.003	[0.999, 1.006]	0.124
Wild-farmed pairs	Intercept	55.27	[48.20, 63.54]	0.000
	Geographic distance	1.000	[0.997, 1.003]	0.992

$Incidence rate ratio;

*Confidence interval
